# Luteolin Attenuates Doxorubicin-Induced Cardiotoxicity Through Promoting Mitochondrial Autophagy

**DOI:** 10.3389/fphys.2020.00113

**Published:** 2020-02-14

**Authors:** Haixia Xu, Wenjun Yu, Shiqun Sun, Congye Li, Yingmei Zhang, Jun Ren

**Affiliations:** ^1^Department of Cardiology, Zhongshan Hospital, Fudan University, Shanghai, China; ^2^Shanghai Institute of Cardiovascular Diseases, Shanghai, China; ^3^Department of Cardiology, Xijing Hospital, Air Force Medical University, Xi’an, China

**Keywords:** luteolin, doxorubicin, cardiotoxicity, mitochondria, autophagy

## Abstract

Doxorubicin is a valuable antineoplastic drug although its clinical use is greatly hindered by its severe cardiotoxicity with dismal target therapy available. Luteolin is a natural product extracted from vegetables and fruits with a wide range of biological efficacies including anti-oxidative, anti-tumorigenic, and anti-inflammatory properties. This study was designed to examine the possible effect of luteolin on doxorubicin-induced cardiotoxicity, if any, and the mechanism(s) involved with a focus on mitochondrial autophagy. Luteolin application (10 μM) in adult mouse cardiomyocytes overtly improved doxorubicin-induced cardiomyocyte contractile dysfunction including elevated peak shortening amplitude and maximal velocity of shortening/relengthening along with unchanged duration of shortening and relengthening. Luteolin alleviated doxorubicin-induced cardiotoxicity including apoptosis, accumulation of reactive oxygen species (ROS) and loss of mitochondrial membrane potential. Furthermore, luteolin attenuated doxorubicin-induced cardiotoxicity through promoting mitochondrial autophagy in association with facilitating phosphorylation of Drp1 at Ser^616^, and upregulating TFEB expression. In addition, luteolin treatment partially attenuated low dose doxorubicin-induced elongation of mitochondria. Treatment of Mdivi-1, a Drp1 GTPase inhibitor, negated the protective effect of luteolin on levels of TFEB, LAMP1, and LC3B, as well as loss of mitochondrial membrane potential and cardiomyocyte contractile dysfunction in the face of doxorubicin challenge. Taken together, these findings provide novel insights for the therapeutic efficacy of luteolin against doxorubicin-induced cardiotoxicity possibly through improved mitochondrial autophagy.

## Introduction

Doxorubicin is an effective anti-neoplastic chemotherapeutic agent although its clinical application has been greatly hindered by its severe and pronounced cardiotoxicity. Ample clinical and experimental evidence has depicted that doxorubicin triggers cardiac anomalies including tachycardia, myocardial injury and heart failure (Sun et al., 2014; Ge et al., 2016; Wenningmann et al., 2019). Over the past decades, extensive efforts have been made toward exploring the mechanisms behind doxorubicin-induced cardiotoxicity including accumulation of reactive oxygen species (ROS), compromised lysosomal function, reduction of ATP production, mitochondrial membrane potential collapse and apoptosis (Octavia et al., 2012; Ma et al., 2017; Guo et al., 2018). Nonetheless, effective target therapy for doxorubicin-induced cardiotoxicity is still lacking.

Luteolin (3′,4′,5′,7′-tetrahydroxyflavone, LUT), as a natural flavone rich in vegetables, fruits and herbs, has shown beneficial properties in multiple biological processes including anti-carcinogenic, anti-apoptotic activities and anti-oxidative stress properties (Rao et al., 2012; Xu et al., 2019). The average consumption of luteolin is approximately 0.01–0.20 mg from our daily diet (Wang et al., 2009). Luteolin is one of the major metabolites upon oral administration of luteolin-7-O-glucoside and generally absorbed by intestinal mucosa into the systemic circulation after oral administration. It was demonstrated that oral bioavailability of luteolin was approximately 26% following administration (Lin et al., 2015). Ample evidence has indicated that luteolin offers cardiovascular protection including ischemia/reperfusion and heart failure through alleviating ROS and apoptosis, as well as intracellular Ca^2+^ dysregulation (Galati et al., 2001; He et al., 2012; Rao et al., 2012; Zhu et al., 2017). Several theories have been proposed for the underlying mechanisms of doxorubicin-induced cardiotoxicity including mitochondrial damage (Marechal et al., 2011; Octavia et al., 2012; Ichikawa et al., 2014), although whether luteolin affects doxorubicin-induced cardiotoxicity remains elusive.

Mitochondria play a pivotal role in energy production, maintaining homeostatic control of ROS production in cardiomyocytes. Clearance of damaged mitochondria exerts a critical role in the mitochondrial quality control. There is emerging evidence for defective mitochondrial autophagy or mitophagy in mitochondrial damage from doxorubicin-induced cardiotoxicity (Abdullah et al., 2019). Mitochondrial autophagy is a conserved cellular process to degrade and recycle damaged mitochondria through formation of autophagosomes and fusion with lysosomes. Autophagy has been shown to play a rather complex role in doxorubicin-induced cardiotoxicity due to excessive or defective autophagy (Li et al., 2016; Koleini and Kardami, 2017; Wang et al., 2019a). It appears that the onset and development of doxorubicin-induced cardiotoxicity is dependent upon drug dosage and duration (Wenningmann et al., 2019). More recent finding has depicted a likely role for interrupted autophagy in low dose doxorubicin (5 mg/kg/week for 4 weeks)-elicited cardiomyopathy (Li et al., 2016). In addition, it was reported that luteolin alleviated post-infarction cardiac dysfunction through upregulation of autophagy (Hu et al., 2016). Given the established property of luteolin on regulation of mitochondrial function and autophagy, this study was designed to examine the impact of luteolin on cardiac contractile function, apoptosis, and mitochondrial autophagy in doxorubicin-induced cardiotoxicity and the underlying mechanisms involved in adult mouse cardiomyocytes (AMCMs).

## Materials and Methods

### Isolation of Adult Murine Cardiomyocytes (AMCMs)

All animal procedures were approved by our institutional Animal Care and Use Committee at the Zhongshan Hospital Fudan University (Shanghai, China). In brief, adult male C57/BL6J mice aged 8 weeks were anesthetized, and chest was opened to fully expose the heart. Inferior vena cava and descending aorta were cut, and EDTA buffer was immediately injected into the right ventricle. Then ascending aorta was tightly clamped and the heart was removed. EDTA buffer and perfusion buffer were injected into left ventricles prior to perfusion of a collagenase buffer (type II and IV collagenase). Left ventricle was then separated and gently pulled into 1 mm^3^ pieces using forceps and was dissociated by gentle pipetting. Cell suspension underwent four sequential rounds of gravity settling, using three intermediate Ca^2+^ reintroduction buffers to gradually restore extracellular Ca^2+^ concentration to 1.2 mM. A yield of at least 80% rod-shaped CMs were deemed successful (Ackers-Johnson et al., 2016; Wang et al., 2018).

### Cell Culture and Treatment

Cardiomyocytes were re-suspended in pre-warmed plating media and plated onto laminin (5 μg/mL) pre-coated culture plastics or glass coverslips in an incubator (37°C, 95% O2, and 5% CO2). Media was changed to replaced 1 h later. After 1 h, AMCMs were treated with DMSO (control), doxorubicin (DOX, 1 μM), luteolin (LUT, at different concentrations of 1, 10, and 50 μM) and the concurrent treatment of doxorubicin and luteolin for 24 h. Mdivi-1 (1 μM) was added in order to confirm the role of Drp1 in luteolin-offered response against doxorubicin-induced cardiotoxicity.

### Measurement of Cell Shortening and Relengthening

Mechanical properties of single cardiomyocytes were measured with an IonOptix Myocam system (IonOptix Inc., Milton, MA, United States). Cells were placed on the stage of an inverted microscope (×400 objective). The cells were stimulated with an electrical field at a frequency of 0.5 Hz. Cell shortening and re-lengthening were assessed according to the previous study with parameters as follows: resting cell length, peak shortening amplitude (PS), time-to-peak shortening (TPS), and maximum velocity of shortening and relengthening (±dL/dt) (Zhang et al., 2014a).

### Assessment of LDH and CK Release

Release of lactate dehydrogenase (LDH) and creatine kinase (CK) was evaluated in culture medium using ELISA assays with the Lactate dehydrogenase assay and Creatine kinase assay kits (Nanjing Jiancheng Bioengineering Institute, Nanjing, China).

### Assessment of TUNEL Staining

Cardiomyocyte apoptosis was analyzed by TUNEL staining using *In Situ* Cell Death Detection Kit (Roche Diagnostics GmbH, Mannheim, Germany). Briefly, after fixed with 4% paraformaldehyde, CMs were incubated with permeabilizing solution for 30 min and were then treated in TUNEL reaction mixture for 1 h at 37°C. Morphological assessment was performed by fluorescence microscopy (20 × objective) (Zhou et al., 2018b). Nine microscopic fields were randomly selected to observe at least 100 cells to assess apoptosis.

### Detection of Reactive Oxygen Species (ROS)

Mitochondrial superoxide level was detected using 2′,7′-dichlorofuorescein-diacetate (DCFH-DA, Beyotime Institute of Biotechnology, Shanghai, China), which can be oxidized by superoxide to emit green fluorescence. Briefly, cells were treated in a ROS working solution for 20 min at 37°C. Thereafter, cells were washed with DMEM three times to remove the dye. A laser confocal microscope microscopy (LECIA) was used to evaluate the fluorescence of ROS production with the Image J software (Zhou et al., 2018a).

### Detection of Mitochondrial Membrane Potential (Δψ*m*)

Δψ*m* was measured using a JC-1 kit (Beyotime Institute of Biotechnology, Shanghai, China) (Zhou et al., 2019). JC-1 forms J-aggregates at high Δψ*m* and emits red fluorescence. However, JC-1 remains in monomer form at low Δψ*m* and emits green fluorescence. AMCMs were stained with a JC-1 solution for 20 min at 37°C according to the manufacturer’s instructions. The ratio of red-to-green fluorescence was calculated using the Image J software to reflect Δψ*m*.

### Immunofluorescence Assay

Immunofluorescence for LC3B and COXIV were performed per the established protocols. Briefly, AMCMs were fixed and permeabilized at room temperature. After blocking for 1 h, AMCMs were incubated with rabbit anti-LC3B antibody (1:50) and mouse anti-COXIV antibody (1:50) overnight at 4°C. Afterward, AMCMs were incubated with anti-rabbit Alexa Fluor 488 (1:800) and anti-mouse Alexa Fluor 546 (1:800 dilution) secondary antibodies for 1 h in the dark (Zhou et al., 2018a). Immunofluorescence was assessed on a laser confocal microscope with a ×630 oil objective (Laser Scanning Confocal Microscopy, Leica, Germany).

### Mitochondrial Isolation and Purification

Mitochondria were isolated from AMCMs using a Mitochondria Isolation Kit (Abcam, ab110170) according to the manufacturer’s instruction. In brief, cells were collected and were homogenized with 30 strokes using pestle B. After centrifuging at 1,000 × *g* for 10 min at 4°C, supernatants were saved. Pellets were resuspended and homogenized. Then, supernatants were combined and were centrifuged at 12,000 × *g* for 15 min at 4°C and were resuspend with a RIPA buffer. Protein concentration was determined using a BCA Protein Assay Kit (Beyotime Institute of Biotechnology, Shanghai, China).

### Western Blot Analysis

Western blot was performed based on our previous report (Zhang et al., 2014b). Cell lysates were extracted in a RIPA buffer supplemented with protease inhibitors. After 20 min, CMs were centrifuged at 12,000 × rpm for 20 min at 4°C, and protein level was determined using a BCA Protein Assay Kit (Beyotime Institute of Biotechnology, Shanghai, China). Samples (25 μg) were analyzed using 10–12% SDS-PAGE, and was then transferred to PVDF membranes. After blocking with 5% non-fat milk, membranes were incubated with primary antibodies overnight at 4°C. Blots were washed three times for 10 min in TBST and were incubated with the HRP-conjugated secondary antibody for 2 h at room temperature. Bands were detected using enhanced chemiluminescence luminal reagents (Bio-Rad Laboratories, United States). Gray value was measured using an Image Lab 3.0 (National Institutes of Health, Bethesda, United States).

### Regents and Antibodies

Doxorubicin (Beyotime Institute of Biotechnology, Shanghai, China), luteolin (≥98%, Santa Cruz Biotechnology, sc-203119), mdivi-1 (≥98%, Sigma Aldrich, M0199). Bax (1:1,000, Cell Signaling Technology, #5023S), Bcl-2 (1:1,000, Cell Signaling Technology, #15071S), Bnip3 (1:1,000, Cell Signaling Technology, #44060), cleaved caspase-9 (1:1,000, Cell Signaling Technology, #9509S), Drp1 (1:1,000, Cell Signaling Technology, #8570), p-Drp1 (Ser^616^) (1:1,000, Cell Signaling Technology, #3455S), LAMP1 (1:500, Abcam, ab208943), LC3B (1:1,000, Abcam, ab48394), mTOR (1:1,000, Cell Signaling Technology, #2983S), p-mTOR (Ser^2448^) (1:1,000, Cell Signaling Technology, #5536S), P62 (1:1,000, Cell Signaling Technology, #5114S), parkin (1:1,000, Cell Signaling Technology, #4211), Pink1 (1:1,000, Abcam, ab216144), TFEB (1:500, Cell Signaling Technology, #32361S), vinculin (1:1,000, Abcam, ab129002);anti-mouse Alexa Fluor (1:1000, Cell Signaling Technology, #4408), anti-rabbit Alexa Fluor (1:1,000, Cell Signaling Technology, #8890);β-actin (1:5,000, KangChen Bio-tech, Shanghai, China).

### Statistical Analysis

Data were reported as mean ± SEM. Statistical analysis was performed using Prism 6.0 software (GraphPad, San Diego, CA, United States). One-way ANOVA followed by Tukey’s test was used to analyze the statistical significance of difference (*P* < 0.05).

## Results

### Luteolin Improved Cardiomyocyte Shortening and Relengthening in the Face of Doxorubicin Challenge

**FIGURE 1:**
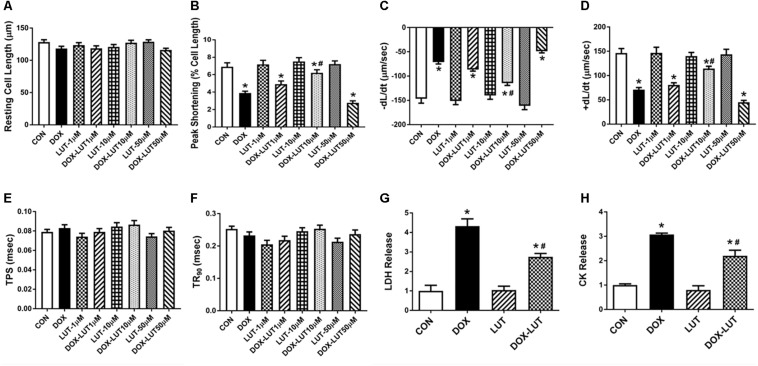
Cardiomyocyte contractile function and cell injury following doxorubicin and luteolin (at various concentrations) exposure for 24 h. **(A)** Resting cell length; **(B)** Peak shortening (PS); **(C)** Maximal velocity of shortening (–dL/dt); **(D)** Maximal velocity of relengthening (+dL/dt); **(E)** Time-to -peak shortening (TPS); **(F)** Time-to-90% relengthening (TR_90_); **(G)** LDH; and **(H)** CK. Mean ± SEM, *n* = 30 cells each group from three independent experiment. * *p* < 0.05 vs. CON group, # *p* < 0.05 vs. DOX group.

To evaluate the effect of luteolin on doxorubicin-induced cardiotoxicity, cell shortening was evaluated in doxorubicin (1 μM, 24 h)-challenged AMCMs in the absence or presence of various concentrations of luteolin (1, 10, and 50 μM). As depicted in Figures 1A–F, doxorubicin treatment overtly decreased peak shortening and ±dL/dt, the effect of which was mitigated by luteolin at the concentration of 10 but not 1 or 50 μM. Luteolin did not exhibit any notable effect itself at these concentrations. Thus, 10 μM was chosen as the concentration for luteolin for the rest of our study, consistent with previous reports (Yao et al., 2016, 2017; Bustos et al., 2018; Park et al., 2018; Zhou et al., 2018b). Neither doxorubicin nor luteolin (at various concentrations), or both, overtly affected resting cell length, TPS and TR_90_ (*P* > 0.05). LDH and CK release were employed to assess cell injuries of cultured AMCMs. Our data shown in Figures 1G,H indicated that doxorubicin overtly promoted release of LDH and CK in cardiomyocytes, the effect of which was significantly attenuated by luteolin at the level of 10 μM.

### Luteolin Attenuated Doxorubicin-Induced Cardiomyocyte Apoptosis

**FIGURE 2:**
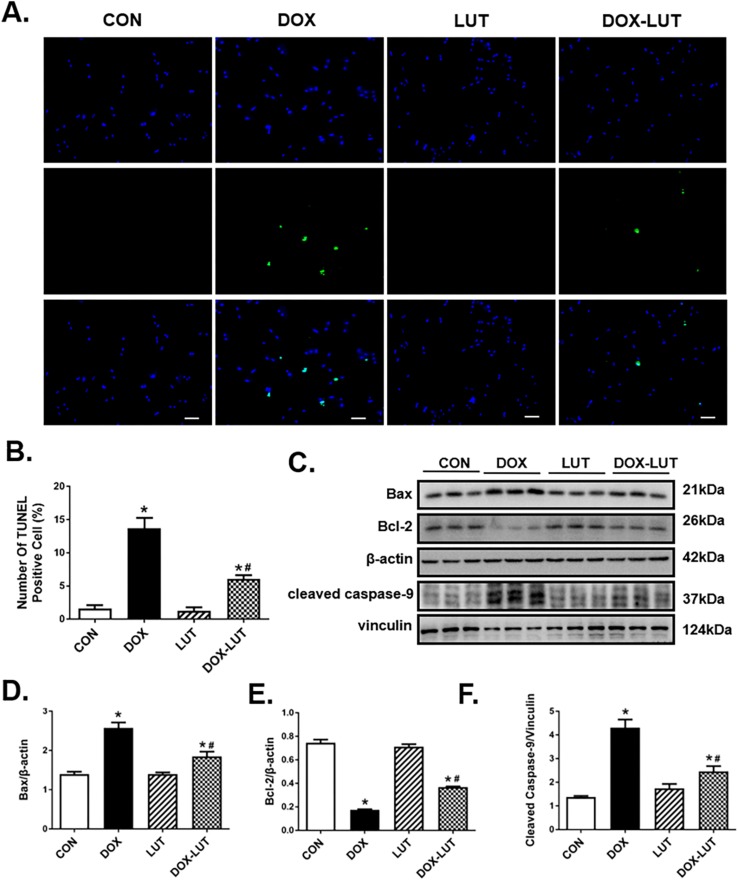
Effect luteolin treatment on doxorubicin-induced cardiomyocyte apoptosis. **(A)** Representative TUNEL staining depicting AMCM apoptosis after doxorubicin and luteolin treatment. All nuclei were stained with DAPI (blue), whereas TUNEL-positive nuclei were visualized using green fluorescence. Original magnification = 200×. **(B)** Quantified TUNEL apoptosis manifested as percentage of TUNEL-positive cells from nine fields per group. **(C)** Representative western blot images of AMCM apoptosis using Bax, Bcl-2 and cleaved caspase-9. **(D–F)** Quantitative analysis of cardiomyocyte apoptosis using Bax, Bcl-2 and cleaved caspase-9. Scale bars = 50 μm. Mean ± SEM, *n* = 3 independent experiment. * *p* < 0.05 vs. CON group, # *p* < 0.05 vs. DOX group.

TUNEL assay was performed to assess cell damage following doxorubicin treatment. In comparison with control group, doxorubicin challenge significantly increased apoptosis as evidenced by the elevated number of TUNEL-positive cardiomyocytes (*P* < 0.01), the effect of which was significantly attenuated by luteolin treatment. Along the same line, Western blot analysis revealed upregulated levels of cleaved caspase-9 and Bax in conjunction with the downregulated Bcl2 levels in doxorubicin-treated AMCMs, the effect of which was partially attenuated by luteolin treatment (Figure 2).

### Luteolin Suppressed Doxorubicin-Induced Mitochondrial Injuries in Cardiomyocytes

**FIGURE 3:**
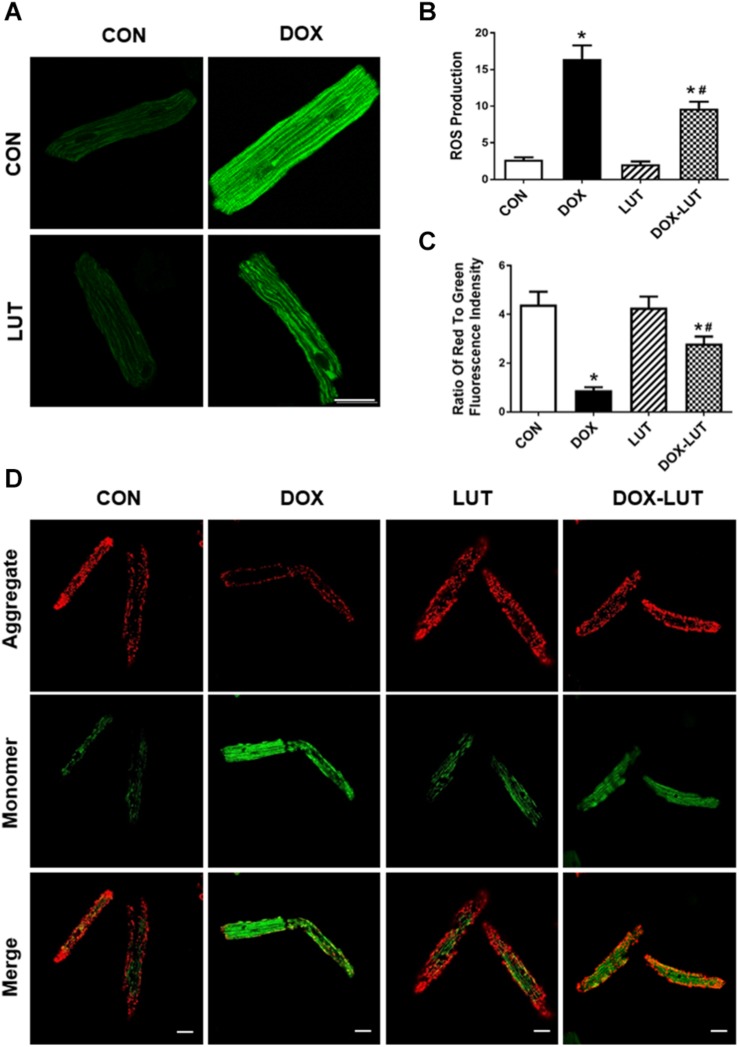
Effect of luteolin on doxorubicin-induced cardiomyocyte mitochondrial injury. **(A)** Representative fluorescence images of AMCMs (original magnification = 630×) showing ROS production in cardiomyocytes after exposure of doxorubicin and luteolin. **(B)** Pooled data of ROS production from nine fields per group. **(C,D)** Representative fluorescence images and quantitative analysis of cardiomyocyte Δψ*m* using JC-1 fluorescence from nine fields (original magnification = 400×). Scale bars = 25 μm. Mean ± SEM, *n* = 3 independent experiments in duplicates per group, * *p* < 0.05 vs. CON group, # *p* < 0.05 vs. DOX group.

Mitochondrial membrane potential and ROS levels were assessed using JC-1 staining and DCF staining, respectively. As shown in Figure 3, doxorubicin treatment overtly decreased ΔΨ*m* and promoted intracellular ROS generation, the effects of which were markedly attenuated by luteolin. Luteolin exerted little effect on ΔΨ*m* and ROS production itself.

### Luteolin Attenuated Doxorubicin-Induced Cardiotoxicity Through Promoting Mitochondrial Autophagy

**FIGURE 4:**
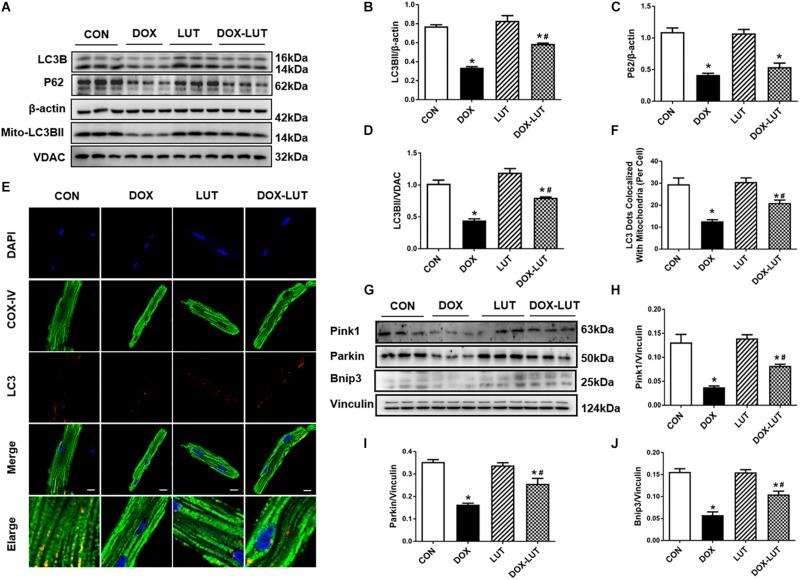
Effect of luteolin treatment on doxorubicin-induced change in cardiomyocyte mitochondrial autophagy. **(A)** Representative Western gel blots depicting protein levels of mitochondrial autophagy, including LC3B and P62 in cardiomyocytes after doxorubicin and luteolin treatment. **(B)** LC3B; **(C)** P62; **(D)** mitochondrial LC3B; **(E)** Representative fluorescence images of LC3B co-localized with mitochondria (COXIV) from 9 to 10 fields per group. **(F)** Quantitative analysis of LC3 dots co-localized with mitochondria. **(G)** Representative Western gel blots of pink1, parkin and Bnip3; **(H)** pink1; **(I)** parkin; **(J)** Bnip3. Scale bars = 25 μm. Mean ± SEM, *n* = 3 independent experiment. * *p* < 0.05 vs. CON group, # *p* < 0.05 vs. DOX group.

To discern the possible role of mitochondrial autophagy following doxorubicin challenge, changes in mitochondrial autophagy protein markers were evaluated. Data in Figures 4A–D indicated that doxorubicin suppressed levels of LC3B, P62 and mitochondrial LC3BII, the effects of which (with the exception of p62) was partially reversed by luteolin. In addition, to further discern the effect of doxorubicin and luteolin on mitochondrial autophagy, we used immunofluorescence technique to evaluate co-localization between LC3B and mitochondria. Mitochondria were labeled with COX-IV (green fluorescence) and LC3B was labeled with red fluorescence, co-localization of mitochondria and LC3B was verified using the merged yellow fluorescence. Our results indicated that doxorubicin overtly decreased the number of LC3 dots co-localized with mitochondria, the effects of which were reversed by luteolin, indicative of improved LC3B abundance in mitochondria in response to luteolin treatment (Figures 4E,F). Moreover, luteolin significantly reversed the inhibition of pink1, parkin and Bnip3 in the face of doxorubicin treatment without eliciting any effect itself (Figures 4G–J).

### Luteolin Promoted Mitochondrial Autophagy Possibly via a Drp1/mTOR/TFEB-Dependent Mechanism

**FIGURE 5:**
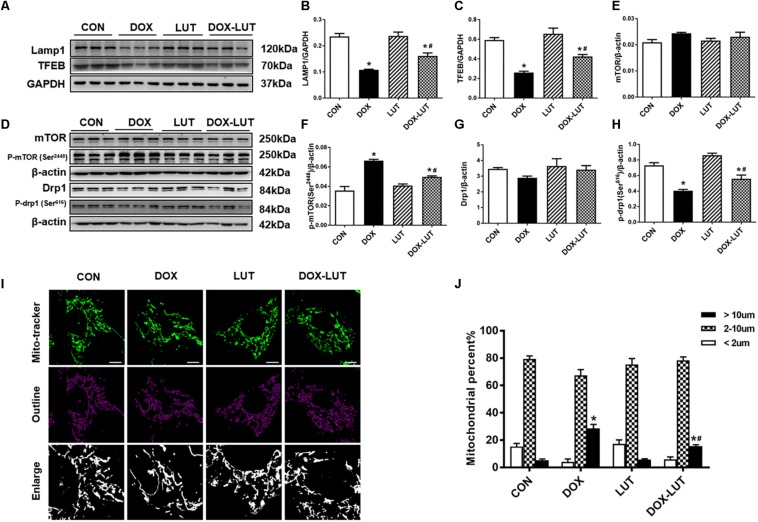
Effect of luteolin treatment on lysosomal Drp1/mTOR/TFEB signaling cascade. **(A)** Representative Western blot of lysosomal generation and number including TFEB and LAMP1. **(B,C)** Quantitative data for TFEB and LAMP1. **(D)** Representative Western blot of mTOR, pSer^2448^-mTOR, Drp1, pSer^616^-Drp1. **(E)** mTOR; **(F)** p-mTOR (Ser^2448^); **(G)** Drp1; **(H)** p-Drp1 (Ser^616^). **(I,J)** Representative fluorescence images and quantitative analysis of morphology of mitochondria in neonatal mouse cardiomyocytes (NMCMs) from 6 fields (original magnification = 630×). Mean ± SEM, *n* = 3 independent experiments in duplicates per group. Scale bars = 10 μm. * *p* < 0.05 vs. CON group, # *p* < 0.05 vs. DOX group.

To explore the possible mechanism of action behind luteolin-promoted autophagy, transcription factor EB (TFEB), a master regulator of autophagy and lysosomal biogenesis (Settembre et al., 2011), were evaluated. As shown in Figures 5A,B, doxorubicin treatment overtly downregulated levels of TFEB, the effect of which was mitigated by luteolin. Given that TFEB is known to regulate lysosomal generation (Settembre et al., 2011), levels of LAMP1 were examined. Our result suggested that doxorubicin downregulated the level of LAMP1, the effect of which was significantly attenuated by luteolin without any notable effect from luteolin itself (Figure 5C).

Since TFEB is negatively regulated through phosphorylation of TFEB by way of mTOR, level of phosphorylated mTOR (Ser^2448^) (Settembre et al., 2012) was examined. Our results indicated that doxorubicin significantly promoted mTOR phosphorylation (Ser^2448^) and inhibited Drp1 phosphorylation (Ser^616^) without affecting pan protein expression of mTOR and Drp1 in AMCMs, the effect of which was overtly attenuated by luteolin (Figures 5D–H). In addition, morphology of mitochondria was scrutinized in neonatal mouse cardiomyocytes (NMCMs). As shown in Figures 5I,J, doxorubicin resulted in elongation of mitochondria, the effect of which was partially attenuated by luteolin treatment.

**FIGURE 6:**
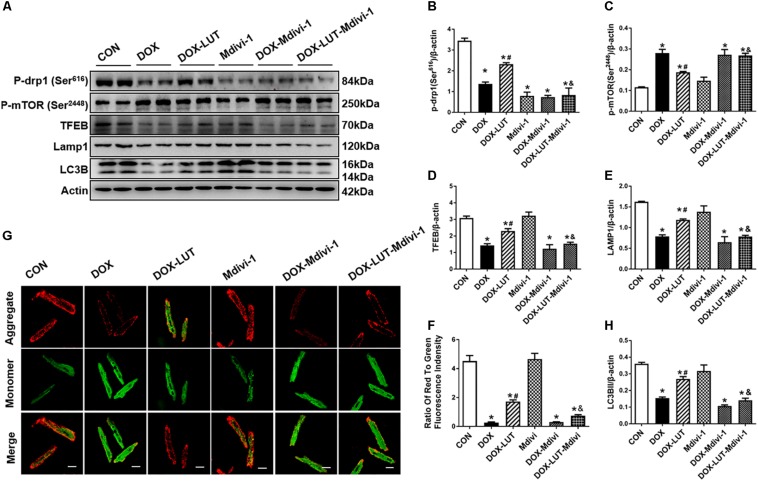
Treatment of Mdivi-1, a Drp1 GTPase inhibitor, on luteolin-offered benefit against doxorubicin-induced cardiotoxicity. **(A)** Representative Western blot of p-Drp1 (Ser^616^), p-mTOR (Ser^2448^), TFEB, LAMP1, and LC3B. **(B)** Quantitative data of p-Drp1 (Ser^616^); **(C)** p-mTOR (Ser^2448^); **(D)** LAMP1; **(E)** TFEB; **(F)** LC3B; and **(G,H)**: Representative fluorescence images and quantitative analysis of cardiomyocyte Δψ*m* using JC-1 fluorescence following mdivi-1 treatment from nine fields. Mean ± SEM, *n* = 3 independent experiments in duplicates per group. Scale bars = 25 μm. * *p* < 0.05 vs. CON group, # *p* < 0.05 vs. DOX group, & *p* < 0.05 vs. DOX-LUT group.

**FIGURE 7:**
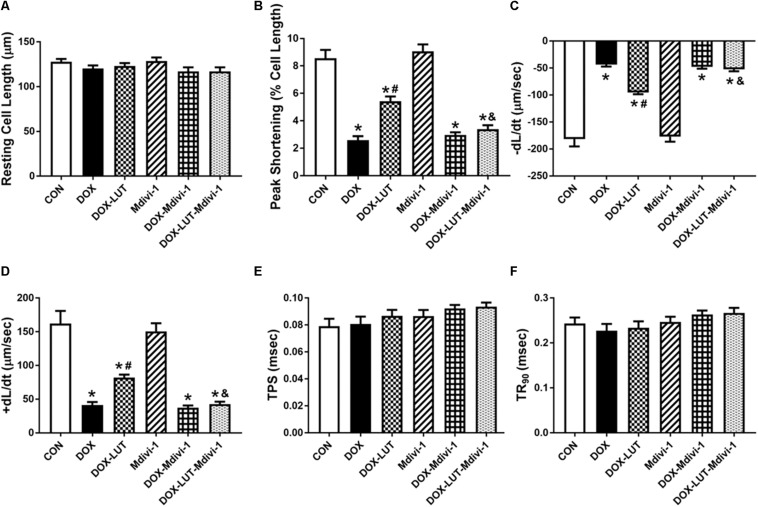
The effect of Mdivi-1 on cardiomyocyte contractile function following doxorubicin and luteolin treatment. **(A)** Resting cell length; **(B)** Peak shortening (PS); **(C)** Maximal velocity of shortening (–dL/dt); **(D)** Maximal velocity of relengthening (+dL/dt); **(E)** Time-to -peak shortening (TPS); **(F)** Time-to-90% relengthening (TR_90_). Mean ± SEM, *n* = 30 cells each group from three independent experiment. * *p* < 0.05 vs. CON group, # *p* < 0.05 vs. DOX group, & *p* < 0.05 vs. DOX-LUT group.

To explore the role of Drp1 in the regulation of mTOR and TFEB in doxorubicin-induced cardiotoxicity, cardiomyocytes were pretreated with mdivi-1, a Drp1 GTPase inhibitor (1 μM for 1 h, prior to exposure of doxorubicin and luteolin for 24 h (Figures 6A–F). Treatment of mdivi-1 obliterated luteolin-offered protective action on levels of LC3B and LAMP1, as well as phosphorylation of mTOR (Ser^2448^) and TFEB. To discern the role of phosphorylated Drp1 (Ser^616^), mitochondrial membrane potential was evaluated using JC-1. Our data shown in Figures 6G,H revealed that treatment of mdivi-1 prevented the protective effect of luteolin on doxorubicin challenge-induced Δψ*m* loss. Moreover, cell shortening was evaluated in AMCMs following mdivi-1 treatment. As shown in Figure 7, mdivi-1 canceled off the beneficial effect of luteolin on doxorubicin-induced decrease in peak shortening and ±dl/dt without eliciting any effect itself in AMCMs.

## Discussion

**FIGURE 8:**
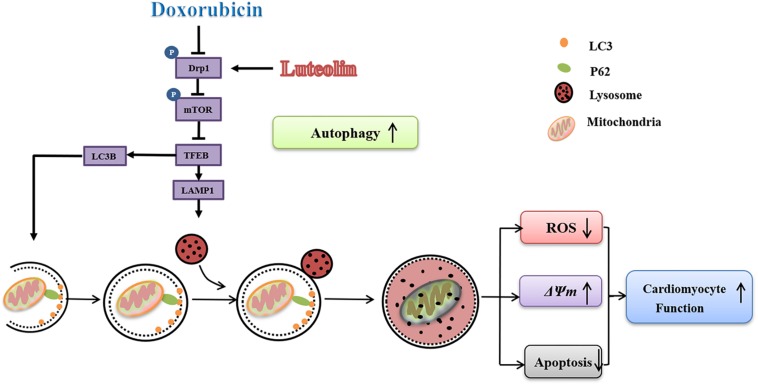
Scheme illustrating proposed possible mechanism of action for luteolin. Doxorubicin challenge inhibits autophagy through disrupting formation of autophagosome and suppressing lysosomal generation in association with changes in Drp1/mTOR/TFEB signaling cascade. Luteolin treatment effectively facilitates autophagosome formation, and improves lysosomal function possibly through a Drp1/mTOR/TFEB-dependent mechanism, resulting in alleviation of ROS accumulation, ΔΨ*m* collapse and apoptosis upon doxorubicin challenge.

The salient findings from our study noted that luteolin protected against doxorubicin-induced contractile dysfunction and apoptosis in cardiomyocytes. Our data also revealed that luteolin attenuated cardiomyocyte mitochondrial injury through regulation of autophagy via Drp1/mTOR/TFEB pathway (Figure 8). Mitochondria-mediated apoptosis plays a pivotal role in programmed cell death (Green, 1998). In this study, doxorubicin decreased the expression of the anti-apoptotic protein Bcl-2, while upregulating levels of pro-apoptotic protein Bax and cleaved caspase-9. These findings were in accordance with previous studies highlighting the pro-apoptotic property of doxorubicin from both *in vivo* and *in vitro* settings (Dimitrakis et al., 2012; Octavia et al., 2012; Bartlett et al., 2016; Li et al., 2016; Wenningmann et al., 2019). An elevated Bax-to-Bcl-2 ratio would disrupt Δψ*m* and activate caspase-9, an apoptotic initiator caspase (Cohen, 1997). In addition, doxorubicin provoked mitochondrial injury including decreased Δψm and increased ROS production, in line with mitochondrial injury noted in doxorubicin-induced cardiotoxicity (Marechal et al., 2011; Octavia et al., 2012; Ichikawa et al., 2014). Treatment of luteolin effectively alleviated doxorubicin-induced mitochondrial apoptosis. Taken together, these findings should help offering novel insights for the therapeutic efficacy of luteolin against doxorubicin-induced cardiomyocyte contractile and mitochondrial defects possibly related to regulation of apoptosis.

Moreover, our finding in AMCMs revealed that doxorubicin treatment interrupted the initiation and completion of mitochondrial autophagy with downregulated levels of TFEB, LC3B, p62, LAMP1, pink1, parkin, and BNIP3. Furthermore, data from our study revealed suppressed TFEB in conjunction with elevated mTOR phosphorylation (Ser^2448^) in doxorubicin-induced cardiotoxicity, in line with previous reports (Settembre et al., 2012; Bartlett et al., 2017; Koleini and Kardami, 2017). It remains debatable whether activating or inhibiting autophagy is beneficial for doxorubicin-induced cardiotoxicity (Sishi et al., 2013; Bartlett et al., 2017; Koleini and Kardami, 2017). It is conceived that low dose doxorubicin disrupts cardiac autophagy by inhibiting lysosomal biogenesis and function due to the abnormalities of TFEB function (Kawaguchi et al., 2012; Bartlett et al., 2016; Wang et al., 2019b). Disruption in cardiac autophagic processes leads to ROS overproduction, and Δψ*m* dissociation, contributing to mitochondria-mediated apoptosis and death, consistent with the previous finding (Bartlett et al., 2016).

Previous Ingenuity Pathway Analysis (IPA) showed that mTOR was one of the proteins which could interact with Drp1 (Chen et al., 2018). In this work, further analysis suggested that Drp1 phosphorylation induced mitochondrial fragmentation, phosphorylation of mTOR and inhibition of autophagic degradation through a Drp1/mTOR/TFEB-dependent pathway (Chen et al., 2018). Up-to-date, few studies have reported the interplay between Drp1 phosphorylation and TFEB. In our study, doxorubicin-induced cardiotoxicity was associated with inhibited phosphorylation of Drp1 at Ser^616^, elevated phosphorylation of mTOR at Ser^2448^ and dampened TFEB expression. In low-dose doxorubicin treated AMCMs, the Drp1 selective inhibitor Mdivi-1 greatly increased phosphorylation of mTOR and decreased TFEB expression. Moreover, it was revealed that low dose of doxorubicin induced elongation of mitochondria in NMCMs. Recent evidence also reported that endogenous Drp1-mediated-mitochondrial autophagy may protect cardiac function in glucose deprivation and pressure overload induced heart failure (Ikeda et al., 2015; Shirakabe et al., 2016). It was indicated that the deletion of Drp1 resulting in mitochondrial elongation and mitochondrial autophagy suppression played a critical role in various cardiac disorders.

Previous analysis showed little changes in Drp1 and more mitochondrial elongation in doxorubicin-treated hearts (Abdullah et al., 2019). It was also suggested that levels of Drp1 and Drp1 Ser^616^ phosphorylation were increased following doxorubicin challenge (Xia et al., 2017; Catanzaro et al., 2019). Indeed, these authors went on to reveal elevated mitochondrial autophagy, contributing to mitochondrial dysfunction and doxorubicin toxicity. Conflicting findings have been noted with either increased or decreased autophagy with either hyperfused or fragmented mitochondria in doxorubicin-induced cardiotoxicity (Lu et al., 2009; Kobayashi et al., 2010; Dimitrakis et al., 2012; Kawaguchi et al., 2012; Sishi et al., 2013). Ample evidence suggested the low-dose doxorubicin was connected with interrupted cardiac autophagy while high-dose doxorubicin was tied with increased autophagy and cardiac dysfunction (Bartlett et al., 2017; Koleini and Kardami, 2017). Clinically, the risk of doxorubicin cardiotoxicity was increased corresponding to the accumulative dose of 400–700 mg/m^2^ and the onset of delayed cardiomyopathy may occur years after initial usage (Koleini and Kardami, 2017). In this report, in order to mimic clinical application of doxorubicin in patients, the observation of experiments was conducted based on a low dose doxorubicin treatment in adult mouse cardiomyocytes which resulted in modest and progressive cardiac injury, and is consistent with conserved study (Minotti et al., 2004; Li et al., 2016). Our data revealed that decreased phosphorylation of Drp1 at Ser^616^ and autophagy upon doxorubicin challenge, which may be due to the low dose doxorubicin induced toxicity in AMCMs.

In this report, our data indicated that luteolin promoted autophagy initiation process and at the same time it promoted the lysosome generation via phosphorylation of Drp1 at Ser^616^ and TFEB, which may become a new therapeutic target of doxorubicin-induced cardiotoxicity due to the restoration of autophagy. It is perceived that a low concentration of luteolin offers cardiac protective effects, including phospholamban phosphorylation, enhance sarcoplasmic reticulum SERCA activity, MAPK signaling pathway and PI3K/Akt-mediated regulations (Wu et al., 2013; Yao et al., 2016; Xia et al., 2017; Wei et al., 2018). Furthermore, pretreatment of luteolin enhances autophagy while 3-methyladenine may block the protective effects of luteolin against myocardial injury induced by starvation (Kawaguchi et al., 2012; Sishi et al., 2013; Yao et al., 2016). In addition, previous work has reported that luteolin significantly attenuated doxorubicin-induced cardiotoxicity by inhibiting ROS accumulation and apoptosis in H9C2 cells (Wang et al., 2010; Yao et al., 2016). This finding suggested potential beneficial effect of luteolin in enhancing cardiac contractility to benefit pathological conditions with compromised heart function including doxorubicin-induced cardiotoxicity. Our present work confirmed protective property of luteolin against doxorubicin-induced cardiotoxicity, possibly related to the capacity of promoting mitochondrial autophagy and improving mitochondrial function.

A number of experimental limitations existed in this study. First and foremost, our study was a cell-based *in vitro* study lacking the *in vivo* proof-of-concept. It would be ideal to use a chronic doxorubicin-induced cardiotoxicity model with intermittent injection of doxorubicin for at least 4 weeks. In addition, it remains elusive with regards to the regulation of luteolin on Drp1 phosphorylation. Possible site of phosphorylation or phosphatase behind luteolin on Drp1 warrants further examination. Meanwhile, the molecular mechanism of Drp1-dependent mitochondrial autophagy remains unclear. In our hands, inhibition of Drp1 phosphorylation might contribute to the activation of mTORC1, the inhibition of TFEB and mitochondrial autophagy in the face of low dose doxorubicin challenge. Further work should focus on the relationship between Drp1 and mTOR, the mechanism of which may partially mediate Drp1-dependent mitochondrial autophagy. Moreover, it still remains uncertain how mTOR phosphorylation contributes to the regulation of mitochondrial autophagy and function. Although our present data seem to favor a role for TFEB-mediated mitochondrial regulation, more in-depth work is needed for the precise mechanism behind mTOR-governed mitophagy response.

In summary, our findings reported that doxorubicin-induced cardiotoxicity is associated with defective mitochondrial autophagy, which leads to mitochondrial dysfunction and cardiomyocyte injury including apoptosis. Luteolin protects cardiomyocytes to some extent via promoting the autophagosome formation and improving lysosomal generation through a Drp1/mTOR/TFEB-dependent mechanism. These findings should provide a new avenue for the treatment of cardiotoxicity in the clinical applications of doxorubicin.

## Data Availability Statement

The datasets generated for this study are available on request to the corresponding author.

## Ethics Statement

The animal study was reviewed and approved by the Zhongshan Hospital Fudan University IACUC.

## Author Contributions

HX, WY, SS, and CL contributed to data collection and data analysis. HX drafted the manuscript. YZ and JR contributed to conception of study, and provided funding and manuscript editing.

## Conflict of Interest

The authors declare that the research was conducted in the absence of any commercial or financial relationships that could be construed as a potential conflict of interest.
